# Terminal S‐Wave Amplitude in Lead V_2_ With Monophasic R Waves in Other Precordial Leads: A Novel ECG Marker for Predicting Acute Successful Endocardial Ablation of a Subtype of Left Ventricular Outflow Tract Idiopathic Ventricular Arrhythmias

**DOI:** 10.1002/clc.70332

**Published:** 2026-04-23

**Authors:** Chengye Di, Longyu Li, Qun Wang, Yanxi Wu, Yan Zhang, Wenhua Lin

**Affiliations:** ^1^ First Department of Cardiology TEDA International Cardiovascular Hospital Tianjin China; ^2^ College of Clinical Cardiology Tianjin Medical University Tianjin China; ^3^ Cardiovascular Institute Tianjin University Tianjin China; ^4^ Chengde Medical University, Chengde Hebei China

**Keywords:** electrogram·radiofrequency catheter ablation, Idiopathic ventricular arrhythmias, left ventricular outflow tract, mapping

## Abstract

**Background:**

An electrocardiographic (ECG) pattern—terminal s‐wave in lead V_2_ with monophasic R waves in other precordial leads—may help identify a subset of left ventricular outflow tract (LVOT) idiopathic ventricular arrhythmias (IVAs) amenable to acute successful endocardial ablation. However, further clinical validation is needed.

**Methods:**

This retrospective study included 93 patients with LVOT IVAs displaying the defined ECG pattern.

**Results:**

Acute successful endocardial ablation was achieved in 70 patients (75.3%; successful endocardial ablation group), while the remaining 23 patients (24.7%; endocardial ablation failure group) required epicardial ablation or had unsuccessful outcomes. The s‐wave amplitude in lead V_2_ was significantly deeper in the successful than in the failure group (5.0 ± 2.6 vs. 2.1 ± 1.1 mV, *p* = 0.000). In the successful group, the most common effective ablation sites were the left/right coronary sinus (LCC/RCC) commissure subvalvularly (52 patients, 55.9%) and anterior mitral annulus near the aorto‐mitral continuity (AMC) (18 patients, 19.4%). In the failure group, success was achieved via the coronary venous system (11 patients), combined endo‐epicardial ablation (3 patients), percutaneous epicardial access or surgical epicardial ablation (3 patients); one procedure failed due to proximity to the left main coronary artery, and 5 patients declined epicardial ablation.

**Conclusions:**

Among patients with this ECG phenotype, approximately 75% can be successfully ablated endocardially at the LCC/RCC commissure subvalvularly or the anterior mitral annulus near the AMC. Greater terminal S‐wave amplitude in lead V_2_ is associated with an increased likelihood of acute endocardial ablation success.

## Graphical abstract

The electrocardiographic (ECG) pattern—terminal s‐wave in lead V_2_ with monophasic R waves in other precordial leads—offers a practical and efficient strategy for electrophysiologists to localize the optimal endocardial target, potentially shortening procedure time, reducing reliance on epicardial access, and enhancing overall ablation success rates.

## 1 Introduction

Idiopathic ventricular arrhythmias (IVAs), including ventricular tachycardia (VT) and premature ventricular contractions (PVCs), frequently originate from the left ventricular outflow tract (LVOT), particularly from the left ventricular summit (LVS)—a site of origin (SOO) that poses technical challenges for radiofrequency catheter ablation due to its complex anatomy and the limitations of current approaches [1, 2]. Despite several proposed electrocardiogram (ECG) criteria for predicting successful endocardial ablation, accurately localizing the SOO still remains difficult [3, 4, 5]. Precise identification of the LVOT IVAs' SOO and optimal endocardial anatomical vantage points for effective ablation is essential for improving outcomes, minimizing repeat procedures, and reducing complications such as coronary artery injury, cardiac pseudoaneurysm, or perforation [6]. Given the similar ECG features shared by LVOT and adjacent regions, distinguishing cases amenable to effective endocardial ablation is challenging. We hypothesized that when the IVAs' SOO is anatomically positioned opposite lead V_2_, the terminal activation vector would be directed away from lead V_2_ and perpendicular to the other precordial leads. We propose that this specific ECG configuration reflects an SOO located near lead V_2_—such as the left/right coronary sinus (LCC/RCC) commissure or the anterior mitral annulus near the aorto‐mitral continuity (AMC)—which are endocardially accessible sites. This study aimed to evaluate this hypothesis.

## 2 Methods

### 2.1 Study Population

Among 635 consecutive outflow tract IVAs patients who presented for ablation between March 2016 and February 2024, 93 LVOT IVAs patients (14.6%) were identified as having ECG characteristics of a terminal s‐wave in lead V_2_ and monomorphic R waves in other precordial leads during episodes of IVAs and were included in this retrospective observational study. All patients had failed at least one anti‐arrhythmic drug. Demographic and clinical data, including patient age, sex, body mass index (BMI), and echocardiography parameters, were collected prior to the index procedure. The study protocol was reviewed and approved by the hospital's ethics committee in accordance with the principles of the Declaration of Helsinki. All patients provided written informed consent before the procedure.

### 2.2 ECG Parameter Assessment, Patient Grouping and Exclusion Criteria

Twelve‐lead ECGs and 24‐h ambulatory Holter monitoring were conducted at least once to assess IVAs morphology and burden before ablation, with ECGs recorded during both sinus beats and clinical IVAs (25 mm/s) using standard chest and limb lead placement, ensuring careful positioning of leads V_1_–V_3_ to avoid alterations in QRS morphology (Libang ECG recording, Shenzhen, China). Two blinded electrophysiologists measured ECG parameters using an electronic caliper; a third resolved discrepancies. IVAs burden was defined as the percentage of IVAs relative to the total number of heartbeats recorded over a 24‐h Holter monitor. Analyzed features included: (1) R/S‐wave amplitudes in leads I, II, III, aVF, V_1_–V_3_; (2) QRS notching; and (3) QRS duration. The T‐P segment served as the baseline. In lead I, R‐wave amplitude was defined as r minus s, positive values indicated r dominance, negative values s dominance. Patients were categorized into the successful endocardial ablation group, defined as those with a terminal s‐wave in lead V_2_ and monomorphic R waves in other precordial leads who were acute successfully ablated endocardially, while the remaining patients formed the endocardial ablation failure group. A monophasic R wave in the precordial leads (excluding V_2_) was defined using the following criteria: (1) Terminal S‐wave amplitude < 0.1 mV; (2) Minor QRS notching was permitted; (3) R/S amplitude ratio > 20. These criteria were applied to ensure reproducibility and objective classification of the ECG morphology. Exclusion criteria: (1) baseline bundle branch block, (2) prior LVOT IVAs ablation, (3) structural heart disease or myocardial infarction. ECG measurements were independently performed by two blinded electrophysiologists, and agreement between observers was evaluated using the intraclass correlation coefficient.

### 2.3 Preparation for Mapping

Antiarrhythmic drugs were stopped ≥ 5 half‐lives before the procedure. Mapping and ablation were performed under local anesthesia with intravenous heparin to maintain activated clotting time at 250–300 s. An irrigated‐tip ablation catheter (TCQ, TCSE [Abbott], or SmartTouch [Biosense‐Webster]) was introduced into the left ventricle (LV) via femoral artery or transseptal access. Standard 12‐lead ECGs and intracardiac electrograms were recorded and stored using the Prucka CardioLab electrophysiology system (General Electric Healthcare, Milwaukee, WI, USA), the Abbott mapping system (Abbott, Minneapolis, USA), or the CARTO3 mapping system (Biosense‐Webster, Diamond Bar, CA, USA) for offline analysis. Bipolar EGMs (distal electrodes) were filtered at 30–500 Hz, unipolar signals at 0.5–100 Hz.

### 2.4 Activation Mapping

Activation mapping was performed during spontaneous IVAs (100 mm/s), recording ≥ 3 beats per site to ensure reproducibility. Activation time was defined as the interval from the earliest bipolar electrogram to QRS onset across 12 ECG leads, using the mean of multiple values. IVAs were induced with intravenous isoproterenol (0.5–2.0 µg/min) if absent or infrequent. Mapping proceeded retrogradely: above and below the aortic valve, then in the distal coronary sinus using an ablation or steerable decapolar catheter. Angiography was used to define the course of the coronary or the great cardiac vein and anterior interventricular vein (GCV/AIV). The SOO was endocardial or epicardial if activation was ≥5 ms earlier at either site, and intramural if < 5 ms difference [5]. Two blinded investigators measured data offline; discrepancies > 5 ms were resolved by consensus.

### 2.5 Pace Mapping

Pace mapping was performed at the earliest activation site using output 1 mA above threshold (max 10 mA, 2.0 ms pulse width). QRS morphology was compared across all 12 leads by evaluating all 12 ECG leads for QRS vector alignment, prominent notching, or baseline deflections. A perfect match was 12/12 lead concordance, excellent was 10–11/12, and a mismatch was < 10/12.

### 2.6 Definition of Acute Successful Endocardial Ablation at the LCC/RCC Commissure Subvalvularly and Anterior Mitral Annulus Near the AMC

Acute successful ablation sites were confirmed using 3D mapping, fluoroscopy, and transthoracic or intracardiac echocardiography (TTE or ICE) imaging. To target the subvalvular LCC/RCC commissure, the catheter was prolapsed retrogradely into the LV and withdrawn with a small curve positioned beneath the commissure along the LV ostium. For ablation at the anterior mitral annulus near the AMC, the catheter was similarly advanced but then decurved with a larger curve to align along the anterior mitral annular near the AMC [7]. Additionally, the annular site near the AMC was confirmed during SR, showed an atrial‐to‐ventricular ratio < 1, with atrial and ventricular amplitudes > 0.08 mV and > 0.5 mV, respectively [8].

### 2.7 Ablation

For endocardial SOO, ablation was done with 35–40 W power, 43°C temperature, and 17 mL/min normal saline irrigation. For intramural SOO, ablation was initially attempted from adjacent endocardial sites—specifically, the subvalvular LCC/RCC commissure or the anterior mitral annulus near the AMC. If ineffective, and the earliest activation was located at the GCV/AIV, coronary venous ablation was performed using 15–20 W. When this approach failed, percutaneous or endoscopic epicardial ablation was considered. The ablation start‐to‐effect time (in seconds) was defined as the time from energy onset to full IVAs suppression. Although acceleration of IVAs may occur during ablation, only complete suppression of the clinical IVAs was used to define the start‐to‐effect time. If suppression didn't occur within 10 s, ablation was stopped; otherwise, it continued for 180–300 s. TTE monitored valve function and complications.

### 2.8 Procedural End Points and Clinical Follow‐Up

Acute success was defined as elimination and non‐inducibility of clinical IVAs after ablation, with or without isoproterenol or pacing, followed by 30–60 min observation. Antiarrhythmic drugs were withheld if ablation succeeded. Patients were monitored with continuous telemetry for at least 2 days post‐procedure, and the twelve‐lead ECG and 24‐h ambulatory Holter monitoring were performed at least once. Follow‐up included outpatient visits or phone calls every 3 months for at least a year. Recurrence was defined as ≥2% IVAs burden on Holter recording within 12 months.

### 2.9 Statistical Analysis

Continuous data were tested for normality (Kolmogorov–Smirnov) and are shown as mean ± SD. Group comparisons used *t*‐tests or rank‐sum tests as appropriate. Categorical data are shown as counts (percentages) and analyzed with the Chi‐square test. Binary logistic regression assessed predictors of endocardial ablation success. Receiver operating characteristic (ROC) analysis evaluated diagnostic performance, with sensitivity, specificity, area under the curve (AUC), and optimal cutoff reported. Statistical significance was defined as *p* < 0.05. To avoid potential multicollinearity between key electrophysiological variables, variance inflation factors (VIF) were calculated to ensure the reliability of the regression analysis. The IBM SPSS 23.0 software (IBM Corporation, New York) was used for analysis.

## 3 Results

### 3.1 Patient Characteristics

Table 1 summarizes the baseline demographic and TTE characteristics of the 93 patients in this study. The successful endocardial ablation group (mean age 60.4 ± 10.8 years) comprised 45 males (64.3%), with hypertension in 23 (32.9%), diabetes in 16 (22.9%), coronary heart disease (CHD) in 22 (31.4%), and a BMI of 26.0 ± 2.7. The endocardial ablation failure group (mean age 69.8 ± 10.5 years) had 15 males (65.2%), hypertension in 10 (43.5%), diabetes in 3 (13.0%), CHD in 3 (13.0%), and a BMI of 27.0 ± 1.7. Except for a significantly higher left ventricular ejection fraction (LVEF) in the successful endocardial ablation group (*p* = 0.018), no other significant differences were observed between the two groups.

**Table 1:** Baseline characteristics of the study population (*N* = 93).

	Successful endocardial ablation group (*N* = 70)	Endocardial ablation failure group (*N* = 23)	*t*/*Z*/*χ* ^2^ value	*p* value
Age, (years)	60.4 ± 10.8*	69.8 ± 10.5*	3.197	**0.001**
IVAs' history, (years)	3.1 ± 3.9*	2.1 ± 1.1*	0.336	0.737
Male sex, *n* (%)	45 (64.3%)	15 (65.2%)	0.007	0.935
Hypertension, *n* (%)	23 (32.9%)	10 (43.5%)	0.853	0.356
DM, *n* (%)	16 (22.9%)	3 (13.0%)	1.026	0.311
CHD, *n* (%)	22 (31.4%)	3 (13.0%)	2.977	0.084
BMI	26.0 ± 2.7	27.0 ± 1.7	1.651	0.102
LVEDD, (mm)	51.6 ± 3.5	53.6 ± 4.0*	1.512	0.131
LVEF, (%)	61.7 ± 4.3	59.0 ± 6.2	2.414	**0.018**

*Note:* Values are given as the mean ± SD or *n* (%) and *indicates non‐normally distributed data. Bold values indicate *p* < 0.05.

Abbreviations: BMI, body mass index; LVEDD, left ventricular end diastolic dimension; LVEF, left ventricular ejection fraction.

### 3.2 ECG Characteristics

**Figure 1:**
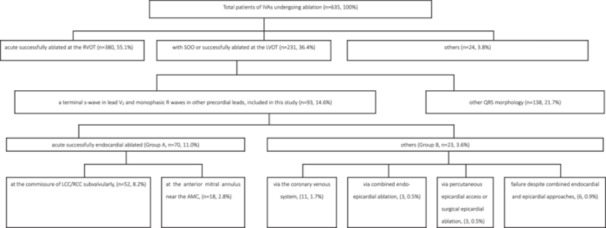
Flowchart of study selection. AMC, aorto‐mitral continuity; IVAs, idiopathic ventricular arrhythmias; LCC/RCC, left/right coronary sinus; LVOT, left ventricular outflow tract; RVOT, right ventricular outflow tract; SOO, site of origin.

Table 2 and Figure 1 presents the ECG features and ablation outcomes. In the successful endocardial ablation group, 64 (91.4%) had monomorphic PVCs and 6 (8.6%) had non‐sustained VTs (defined as three or more consecutive PVCs), while in the endocardial ablation failure group, 19 (82.6%) had monomorphic PVCs and 4 (17.4%) had non‐sustained VTs. No S‐waves were observed in leads V_4_–V_6_. The terminal s‐wave in lead V_2_ was significantly deeper in the successful endocardial ablation group (5.0 ± 2.6 vs. 2.1 ± 1.1 mV, *p* = 0.000), while extension into lead V_3_ was similar between groups (25.7% vs. 39.1%, *p* = 0.930). The successful endocardial ablation group had shorter QRS durations (149.9 ± 14.9 vs. 193.2 ± 25.6 ms, *p* < 0.001), higher R‐wave amplitudes in lead I (–0.25 ± 2.6 vs. –3.3 ± 3.3 mV, *p* < 0.001) and lead II (18.4 ± 4.6 vs. 14.8 ± 3.2 mV, *p* = 0.001), but not in lead III (*p* > 0.05). IVAs burden was comparable (22.2 ± 9.6% vs. 25.6 ± 10.7%, *p* = 0.189).

**Table 2:** Electrocardiogram, electrophysiological characteristics, and ablation result of the study population (*N* = 93).

	Successful endocardial ablation group (*N* = 70)	Endocardial ablation failure group (*N* = 23)	t/Z/χ2 value	*p* value
QRS duration during IVAs, (ms)	149.9 ± 14.9*	193.2 ± 25.6	6.042	**< 0.001**
IVAs burden, (%)	22.2 ± 9.6*	25.6 ± 10.7*	1.314	0.189
Clinical IVAs			0.635	0.426
Only PVC, *n* (%)	64 (91.4%)	19 (82.6%)		
PVC + non‐sustained VT, *n* (%)	6 (8.6%)	4 (17.4%)		
Lead I amplitude	−0.25 ± 2.6*	−3.3 ± 3.3*	4.463	**< 0.001**
Lead V_1_ R wave amplitude, (mV)	7.0 ± 3.4*	7.7 ± 2.2	1.588	0.112
Lead V_2_ R wave amplitude, (mV)	16.3 ± 7.7	16.7 ± 4.2*	1.016	0.310
Lead V_2_ s wave amplitude, (mV)	5.0 ± 2.6*	2.1 ± 1.1	4.947	**0.000**
Lead V_3_ s wave amplitude, (mV)	0.82 ± 0.42*▽	0.92 ± 0.63*▼	0.088	0.930
Lead II R wave amplitude, (mV)	18.4 ± 4.6*	14.8 ± 3.2	3.456	**0.001**
Lead III R wave amplitude, (mV)	18.4 ± 4.5	16.5 ± 3.1*	1.843	0.065
Earliest endocardial bipolar V‐QRS interval, (ms)	−27.9 ± 9.4	−15.8 ± 6.7*	5.064	**< 0.001**
Earliest epicardial bipolar V‐QRS interval, (ms)	−8.8 ± 8.7△	−22.5 ± 12.5▲	5.618	**< 0.001**
Initial QS‐shaped unipolar electrograms, *n* (%)	53 (75.7%)	17 (73.9%)	0.030	0.862
Perfect (12/12 leads) pace mapping, *n* (%)	9 (12.9%)	7 (30.1%)	2.622	0.105
RFCA start to effect time, (s)	6.2 ± 2.5@	7.4 ± 2.5#	1.637	0.105
Follow up period, (month)	24.1 ± 11.6*	27.5 ± 10.5	1.523	0.128
Recurrence during the follow up, *n* (%)	6 (8.6%)	8 (34.8%)	7.364	**0.007**

*Note:* Values are given as the mean ± SD or *n* (%) and *Indicates non‐normally distributed data. Bold values indicate *p* < 0.05. @indicate data for the 64 patients (91.4%) in successful endocardial ablation group and #indicate data for the 14 patients (60.9%) in the endocardial ablation failure group, in the remaining patients, the duration of successful RFCA time was not well determined due to the infrequent nature of clinical VAs during ablation. ▽indicate data for the 18 patients (25.7%) in successful endocardial ablation group and ▼indicate data for the 9 patients (39.1%%) in the endocardial ablation failure group, in the remaining patients the terminal s‐wave were only present in lead V_2_. △indicate data for the 61 patients (87.1%) in successful endocardial ablation group and ▲indicate data for the 17 patients (73.9%) in endocardial ablation failure group, in the remaining patients, the earliest epicardial bipolar V‐QRS interval was not available due to unable to advance or position the catheter to the distal portion of the coronary sinus or refusal to undergo percutaneous or endoscopic epicardial ablation.

### 3.3 Endocardial and Epicardial Activation Mapping Differences

Simultaneous endocardial and epicardial mapping was performed in 88 patients (94.6%), while 5 (5.4%) had only endocardial mapping due to coronary venous inaccessibility or refusal of epicardial access. The earliest endocardial bipolar V–QRS interval was significantly longer in the successful endocardial ablation group (–27.9 ± 9.4 ms vs. –15.8 ± 6.7 ms, *p* < 0.001), while the earliest epicardial V–QRS interval was shorter (–8.8 ± 8.7 ms vs. –22.5 ± 12.5 ms, *p* < 0.001). In the successful endocardial ablation group, the IVAs' SOO were endocardial in 16 (17.2%), intramural in 30 (32.3%), and epicardial in 24 (25.8%). In the endocardial ablation failure group, it was intramural in 4 (13.8%) and epicardial in 14 (73.1%); no endocardial SOO was observed (Table 2). The remaining 5 patients (5.4%) underwent only endocardial mapping due to the same limitations mentioned above. QS‐shaped unipolar EGMs were similar between groups (75.7% vs. 73.9%, *p* = 0.862).

### 3.4 Pace Mapping Results

A perfect pace mapping result was obtained at the successful ablation site only in 9 patients (12.9%) in the successful endocardial ablation group and 7 patients (30.1%) in the endocardial ablation failure group (*p* = 0.105). Additionally, 1 patient (1.1%) in the successful endocardial ablation group exhibited perfect pace mapping with a stimulus‐QRS interval of 28 ms to the onset of the QRS complex. In the remaining 71 patients (76.3%), a mismatch pace‐mapping result was observed at the successful ablation site.

### 3.5 Ablation Outcomes

In the successful endocardial ablation group, the main targets were the LCC/RCC commissure subvalvularly (74.3%) and the anterior mitral annulus near the AMC (25.7%). In the endocardial ablation failure group, ablation was successful via the coronary venous system (11 patients), combined endo‐epicardial approach (3 patients), or epicardial access (3 patients); one case failed due to proximity to the left main coronary artery, and five declined epicardial ablation. The start‐to‐effect time was 6.2 ± 2.5 s in 64 patients (91.4%) in the successful endocardial ablation group and 7.4 ± 2.5 s in 14 patients (60.9%) in the endocardial ablation failure group. In the remaining patients, timing data were unavailable due to technical or procedural limitations. Multivariable logistic regression identified the earliest endocardial bipolar V–QRS interval (OR = 1.333, *p* = 0.044), lead V_2_ s‐wave amplitude (OR = 6.037, *p* = 0.027), and QRS duration (OR = 0.888, *p* = 0.030) as independent predictors of acute successful endocardial ablation (Table 3). Multicollinearity among key electrophysiological variables (QRS duration, V_2_ S‐wave amplitude, and V_2_ R‐wave amplitude) was assessed using VIF. The VIF values were 1.28, 1.07, and 1.23, respectively. To evaluate the incremental contribution of V_2_ S‐wave amplitude beyond QRS duration, an additional binary logistic regression model including both variables was performed. After adjustment for QRS duration, V_2_ S‐wave amplitude remained significantly associated with acute successful endocardial ablation (OR = 1.509, 95% CI: 1.005–2.266, *p* = 0.047) (Table 4). The Hosmer–Lemeshow goodness‐of‐fit test demonstrated acceptable model calibration (*χ*² = 7.107, *p* = 0.525). Model performance metrics included Nagelkerke *R*² = 0.363, indicating good explanatory power. ROC analysis showed that a V_2_ S‐wave amplitude > 2.6 mV predicted successful endocardial ablation with an AUC of 0.844 (*p* < 0.001), sensitivity of 87.1%, and specificity of 77.1%, demonstrating good discrimination.

**Table 3:** Binary logistic regression model for the prediction of successfully endocardial ablation of IVAs (*n* = 93).

Variables	*B*	SE	Wald	*p* value	OR	95% CI
Age, (years)	−0.111	0.079	1.964	0.161	0.895	0.767−1.045
Sex	−1.866	1.780	1.100	0.294	0.155	0.005–5.064
Earliest endocardial bipolar V‐QRS interval, (ms)	0.288	0.143	4.066	**0.044**	1.333	1.008−1.763
Ablation start to effect time, (s)	−0.468	0.296	2.501	0.114	0.626	0.351–1.118
Lead V_2_ R‐wave amplitude, (mV)	−0.203	0.153	1.765	0.184	0.816	0.605–1.101
Lead V_2_ s‐wave amplitude, (mV)	1.798	0.814	4.881	**0.027**	6.037	1.225–29.752
QRS notch	0.433	1.799	0.058	0.810	1.541	0.045–52.537
QRS duration, (ms)	−0.119	0.055	4.707	**0.030**	0.888	0.797–0.989
Pace mapping result	−0.138	2.030	0.005	0.946	0.871	0.016–46.579

*Note:* Bold values indicate *p* < 0.05.

Abbreviations: CI, confidence interval; OR, odds ratio.

**Table 4:** Binary logistic regression model for the prediction of whether V2 S‐wave adds predictive value after accounting for QRS duration (*n* = 93).

Variables	*B*	SE	Wald	*p* value	OR	95% CI
QRS duration, (ms)	−0.029	0.011	7.486	**0.006**	0.971	0.951–0.992
Lead V_2_ s‐wave amplitude, (mV)	0.411	0.207	3.934	**0.047**	1.509	1.005–2.266

*Note:* Bold values indicate *p* < 0.05.

Abbreviations: CI, confidence interval; OR, odds ratio.

### 3.6 Observations after Ablation and during the Follow‐Up

Three groin hematomas occurred post‐procedure, but no major complications (e.g., valve injury, embolism, coronary damage, or pericardial effusion) were detected by ECG, angiography, or TTE. During the 2‐day hospital stay, IVAs recurred in 1 patient (1.4%) in the successful endocardial ablation group and 2 (8.7%) in the endocardial ablation failure group. Over 24.1 ± 11.6 months of follow‐up (3 lost), 6 patients (8.6%) in the successful endocardial ablation group had IVAs recurrence; 2 underwent re‐do ablation at the same targets as those in the index procedure, as confirmed by X‐ray fluoroscopic views, ICE, and three‐dimensional mapping results. In the endocardial ablation failure group, over 27.5 ± 10.5 months (1 lost), 8 patients (34.8%) had recurrence; 3 had re‐do ablation—2 at the same targets as those in the index procedure and 1 at the subvalvular LCC/RCC commissure.

## 4 Discussion

### 4.1 Main Findings

This study highlights three key findings: (1) Among 635 patients with IVAs, 93 (14.6%) exhibited a characteristic ECG pattern of a terminal s‐wave in lead V_2_ and monophasic R waves in other precordial leads, defining a distinct LVOT IVAs subgroup. (2) Within this phenotype, seventy of these patients (75.3%) achieved acute successful endocardial ablation, and greater terminal S‐wave amplitude in lead V_2_ was associated with an increased likelihood of acute endocardial ablation success. (3) The LCC/RCC commissure subvalvularly, followed by the anterior mitral annulus near the AMC, were the most effective targets.

### 4.2 ECG Characteristics of LVOT IVAS Successfully Ablated Endocardially

**Figure 2:**
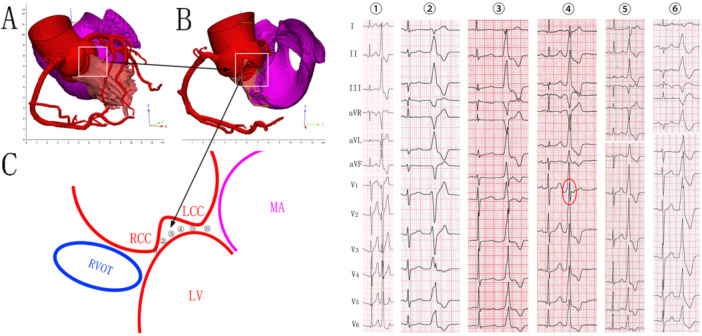
Anatomy of the left ventricular summit (LVS) and representative 12‐lead electrocardiograms (ECGs) of idiopathic ventricular arrhythmias (VAs) originating from LVS regions. A–C: Illustrations show the confined, fan‐shaped region of the LVS and the anatomical alignment of the LCC/RCC commissure and the anterior mitral annulus near the AMC in an anteromedial to posterolateral direction in the transverse view. Representative 12‐lead ECGs show characteristic patterns corresponding to varying SOO: (1) qrS patterns in leads V_1_ and V_2_ with late transition suggest an SOO at the most septal portion of the LCC/RCC commissure; (2) late precordial transition at lead V_4_ without QRS notching in leads V_1_ and V_2_ indicates a slightly leftward SOO from the septal portion of the LCC/RCC commissure; (3) precordial R/S > 1 transition at lead V_1_, accompanied by terminal s‐waves in leads V_1_ and V_2_ but without QRS notching in inferior leads, suggests a further leftward SOO; (4) the distinct ECG pattern characterized in this study—narrower QRS duration and absence of QRS notching in inferior leads—indicates a SOO anatomically located beneath lead V_2_; (5) monomorphic R waves in all precordial leads without QRS notching in inferior leads suggest a more posterior‐leftward SOO; (6) monomorphic R waves in all precordial leads with accompanying QRS notching in inferior leads indicate the most superior‐posterior‐leftward SOO.

**Figure 3:**
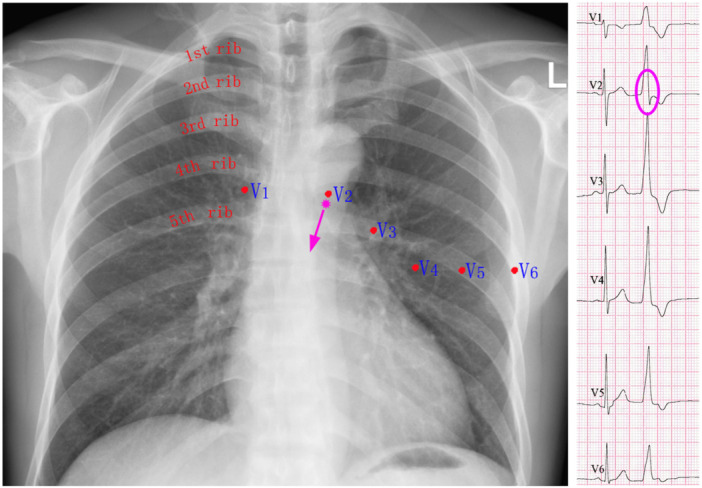
Proposed mechanism for the genesis of the terminal s‐wave in lead V_2_. In IVAs originating from the LCC/RCC commissure or the anterior mitral annulus near the AMC, the distal RVOT remains the latest activated region. This delayed activation generates a terminal depolarization vector directed away from lead V_2_ and perpendicular to the other precordial leads, resulting in a terminal s‐wave in lead V_2_.

**Figure 4:**
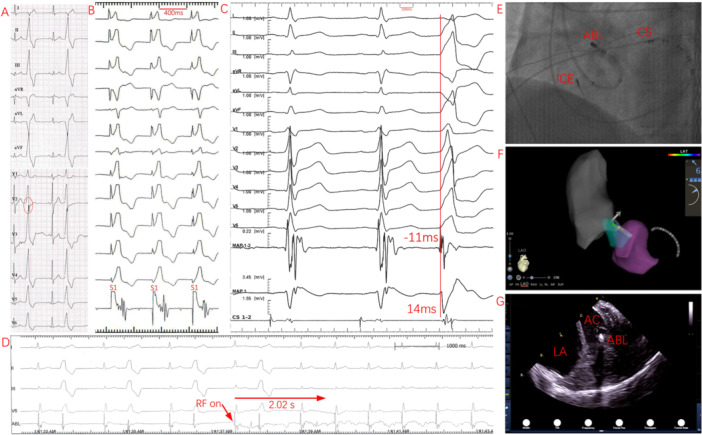
PVC acutely ablated at the LCC/RCC commissure subvalvularly. (A) Twelve‐lead ECG showing a terminal s‐wave in lead V_2_. (B) Pace mapping demonstrated a mismatch with the clinical PVC. (C) Simultaneous endocardial and epicardial mapping revealed the earliest endocardial bipolar V–QRS interval of –11 ms with an initial rS‐shaped unipolar electrogram, while the earliest epicardial bipolar V–QRS interval was 14 ms. (D) Immediate cessation of IVAs was observed following successful ablation within 2.02 s. (E, F) Left anterior oblique fluoroscopy and 3D electroanatomic mapping confirmed that the ablation catheter, introduced retrogradely into the LV, was positioned at the LCC/RCC commissure subvalvularly using a small‐curve configuration. (G) Although ICE can provide short‐axis views of the aortic root, it is unable to simultaneously visualize both the LCC/RCC commissure and the subvalvular catheter tip. ABL, ablation catheter; AC, aortic cusp; CS, coronary sinus catheter; ICE, intracardiac echocardiography; LA, left atrium.

**Figure 5:**
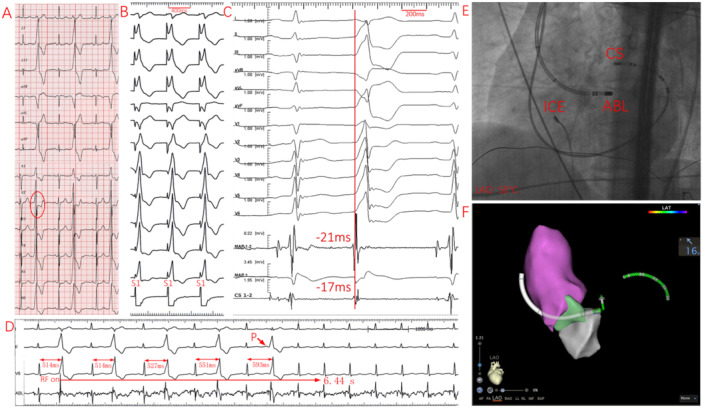
PVC acutely ablated at the anterior mitral annulus near the AMC. (A) Twelve‐lead ECG showing a characteristic terminal s‐wave in lead V_2_. (B) Pace mapping demonstrated a mismatch with the clinical PVC. (C) Simultaneous endocardial and epicardial mapping revealed the earliest endocardial bipolar V–QRS interval of –21 ms with an initial QS‐shaped unipolar electrogram, and the earliest epicardial bipolar V–QRS interval of –17 ms. The ablation target was further confirmed during SR by a typical annular electrogram, with an atrial‐to‐ventricular amplitude ratio of 0.33 (atrial amplitude: 0.28 mV; ventricular amplitude: 0.85 mV). (D) Progressive delay in PVC coupling interval was observed—from 514 ms pre‐ablation to 527 ms, 551 ms, and 593 ms—followed by complete elimination of IVAs within 6.44 s after ablation. (E, F) Left anterior oblique fluoroscopy and 3D electroanatomic mapping confirmed catheter placement at the anterior mitral annulus near the AMC using a large‐curve configuration. ICE could not visualize the ablation catheter location due to its inability to provide short‐axis views of the mitral annulus.

The LV ostium, the most superior part of the LVOT, typically produces IVAs with cranio‐caudal activation and an inferior QRS axis [9]. The LCC/RCC commissure (anteromedial) and anterior mitral annulus near the AMC (posterolateral) define the transverse plane of the LV ostium. This orientation shapes precordial ECG patterns that help localize IVAs' SOO and guide ablation (Figure 2) [5, 10, 11]: (1) notched, “W,” or qrS patterns in lead V_1_/V_2_ with late transition suggest a typically SOO at the most septal portion of the LCC/RCC commissure [3]; (2) as the SOO shifts slightly leftward, late precordial transition with an abrupt V_3_ transition pattern emerges [5]; (3) further shifting in the same direction results in a precordial R/S > 1 transition before lead V_2_, usually accompanied by a terminal s‐wave in lead V_1_/V_2_ but without a QRS notch; (4) IVAs with a terminal s‐wave in lead V_2_ and monophasic R waves in other precordial leads –a less common and unique ECG pattern as shown in this study, typically having a narrower QRS duration and absence of QRS notches due to their relative septal SOO (Figure 3, Figure 4 and Figure 5); (5) as the SOO moves even further posterior‐leftward, monomorphic R waves appear in all precordial leads, with or without a QRS notch; (6) at the most superior‐posterior‐leftward SOO, monomorphic R waves in all precordial leads are accompanied by a QRS notch [12, 13]. Minor SOO shifts within this confined, fan‐shaped subvalvular LVOT region can significantly alter ECG patterns. Furthermore, overlap of anatomical structures and conduction variability—such as preferential pathways or multiple exits—can obscure localization, affecting ablation success and risk [12].

### 4.3 Anatomic Considerations and Proposed Mechanism for the Genesis of Terminal S‐Wave in Lead V_2_

During SR, the final ventricular activation occurs at the LV base or right ventricular outflow tract (RVOT), producing a terminal vector directed away from lead V_2_ and resulting in a terminal s‐wave [14, 15]. Similarly, in IVAs arising from the LCC/RCC commissure or anterior mitral annulus near the AMC—structures located just beneath lead V_2_—the distal RVOT remains the latest to activate, generating a terminal vector away from lead V_2_ and perpendicular to other precordial leads. This explains the presence of a terminal s‐wave in lead V_2_ and monophasic R waves elsewhere (Figure 2 and 3). Therefore, this ECG pattern indicates a SOO near the LCC/RCC commissure or anterior mitral annulus near the AMC—structures anatomically located just beneath lead V_2_. Occasionally, this vector extends to lead V_3_, as observed in 18 patients (25.7%) in the successful endocardial ablation group and 9 (39.1%) in the endocardial ablation failure group (Table 2).

### 4.4 Vantage Points for Acute Successful Endocardial Ablation of LVOT IVAs

**Figure 6:**
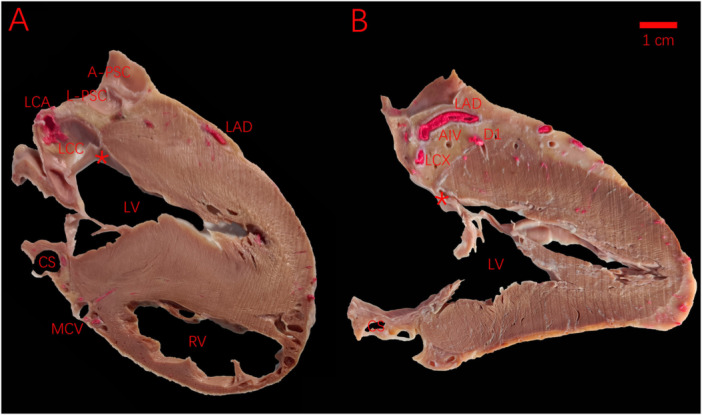
Gross pathological examination reveals that the LV myocardium tapers as it attaches to the aortic and mitral annuli, with a median thickness of 4–5 mm at the LV ostium (marked as *). (A) Non‐standard section at the LCC/RCC commissure showing the LCC, LCA, L‐PSC and A‐PSC. (B) Non‐standard section perpendicular to the mitral annulus and parallel to the ventricular septum. This anatomical configuration facilitates effective transmural lesion formation via endocardial ablation, enabling the successful elimination of intramural or epicardial IVAs. AIV, anterior interventricular vein; A‐PSC, anterior pulmonary sinus cusp; CS, coronary sinus; D1, first diagonal branch; LAD, left anterior descending artery; LCA, left coronary common artery; LCC, left coronary cusp; LCX, left circumflex artery; L‐PSC, left pulmonary sinus cusp; LV, left ventricle; MCV, middle cardiac vein; RV, right ventricle;

Gross pathological examination reveals that the LV myocardium tapers as it attaches to the aortic and mitral annuli, with a median thickness of 4–5 mm at the LV ostium (Figure 6). Microscopic analysis has demonstrated that lesion diameters of 5–8 mm can be achieved through endocardial ablation [5]. These findings confirm the efficacy of the endocardial approach in producing transmural lesions capable of eliminating IVAs with tramural or epicardial SOO. Compared to the coronary venous route, endocardial ablation allows safer, high‐power energy delivery with lower risk of collateral injury [16]. Some cases may need ablation from multiple sites. In this study, the endocardial strategy—unlike the epicardial approach—was not associated with any acute or long‐term complications, highlighting the LCC/RCC commissure and anterior mitral annulus near the AMC as optimal vantage points for effective ablation of IVAs characterized by the defined ECG pattern [17].

### 4.5 Anatomic Considerations for Effective Subvalvular IVAS Ablation

Although IVAs can theoretically be approached from either side of the aortic valve, the LV ostial myocardium is the key target [4, 18]. Ablation from above the valve (within the coronary cusps or commissure) must penetrate fibrous tissue, limiting lesion depth. In contrast, subvalvular ablation at the LCC/RCC commissure allows direct contact with the tapered myocardium, improving efficacy. In this study, ablation via a reversed U‐curve was more effective when targeting this site. For deeper SOOs, advanced techniques—combined endo‐epicardial ablation, bipolar delivery, half‐normal saline, or ethanol infusion—may be needed [19, 20, 21, 22, 23]. Due to anatomic proximity, ablation at slightly suboptimal sites near the LCC/RCC commissure or AMC may still succeed [24, 25, 26]. Among the successful endocardial ablation group, 52 (74.3%) were treated at the LCC/RCC commissure subvalvularly and 18 (25.7%) at the anterior mitral annulus near the AMC, with 89% arrhythmia‐free survival over 25 ± 11 months.

### 4.6 Study Limitations

This study has several limitations. First, it included only patients without structural heart disease, limiting applicability to those with structural abnormalities. Second, QRS morphology may be influenced by anatomical variation, body habitus, hypertrophy, and preferential conduction, which could affect interpretation. Third, the anatomical localization of successful ablation sites was based on fluoroscopy, TTE, or ICE. While ICE can provide short‐axis views of the aortic root, it is rarely able to simultaneously visualize both the LCC/RCC commissure and the subvalvular catheter tip as effectively as TEE; however, TEE was not used due to patient discomfort. Fourth, the choice and sequence of ablation targets were not standardized and depended on operator discretion. Fifth, the proposed vector explanation linking the terminal S‐wave in lead V_2_ to delayed distal RVOT activation remains hypothetical, as direct activation mapping evidence was not available in the present study. Further studies are needed to validate these findings and assess their broader clinical relevance.

## 5 Conclusions

Among patients with this ECG phenotype, approximately 75% can be successfully ablated endocardially at the LCC/RCC commissure subvalvularly or the anterior mitral annulus near the AMC. Greater terminal S‐wave amplitude in lead V_2_ is associated with an increased likelihood of acute endocardial ablation success. This ECG pattern offers a practical and efficient strategy for electrophysiologists to localize the optimal endocardial target, potentially shortening procedure time, reducing reliance on epicardial access, and enhancing overall ablation success rates.

## Author Contributions

**Chengye Di:** conceptualization, methodology, reviewing, and editing the manuscript. **Longyu Li** and **Qun Wang:** writing discussion, abstract, manuscript submission. **Yanxi Wu** and **Yan Zhang:** writing results, supporting materials. **Wenhua Lin:** introduction, graphical abstract. **Chengye Di:** data curation, manuscript editing. **Wenhua Lin:** conceptualization, supervision, critical revision of the manuscript, final approval.

## Conflicts of Interest

The authors declare no conflicts of interest.

## Data Availability

The data sets used and analyzed during the current study are available from the corresponding author on reasonable request.
